# 5-Nitrothiazol-Clofibric Acid Analogs as Potent Agents Against Nitazoxanide-Resistant *Giardia lamblia*: Modulation of Glycolytic Genes and Molecular Dynamics Studies

**DOI:** 10.3390/molecules31172980

**Published:** 2026-08-26

**Authors:** Beatriz Hernández-Ochoa, Blanca Colin-Lozano, Gabriel Navarrete-Vázquez, Sergio Enríquez-Flores, Ignacio De la Mora-De la Mora, Verónica Pérez de la Cruz, Roberto Arreguin-Espinosa, Rosa Angélica Castillo-Rodríguez, José Antonio Velázquez-Aragón, Julieta Griselda Mendoza-Torreblanca, Noemí Cárdenas-Rodríguez, Daniel Ortega-Cuellar, Cindy Bandala, Abraham Vidal-Limon, Saúl Gómez-Manzo

**Affiliations:** 1 Laboratorio de Investigación en Ciencias Ómicas y Epidemiología Microbiana, Hospital Infantil de México Federico Gómez, Secretaría de Salud, Mexico City 06720, Mexico; beatrizhb_16@ciencias.unam.mx; 2 Departamento de Química, Universidad La Salle, Mexico City 06140, Mexico; blanca.colin@lasalle.mx; 3 Facultad de Farmacia, Universidad Autónoma del Estado de Morelos, Cuernavaca 62209, Morelos, Mexico; gabriel_navarrete@uaem.mx; 4 Laboratorio de Biomoléculas y Salud Infantil, Instituto Nacional de Pediatría, Secretaría de Salud, Mexico City 04530, Mexico; sergioenriquez@ciencias.unam.mx (S.E.-F.); ignaciodelamora@ciencias.unam.mx (I.D.l.M.-D.l.M.); 5 Neurobiochemistry and Behavior Laboratory, National Institute of Neurology and Neurosurgery “Manuel Velasco Suárez”, Mexico City 14269, Mexico; veronica.perez@innn.edu.mx; 6 Departamento de Química de Biomacromoléculas, Instituto de Química, Universidad Nacional Autónoma de México, México City 04510, Mexico; arrespin@unam.mx; 7 Centro de Investigación en Ciencia Aplicada y Tecnología Avanzada (CICATA) Unidad Morelos, Instituto Politécnico Nacional, Xochitepec 62790, Morelos, Mexico; racastillo@ipn.mx; 8 Laboratorio de Oncología Experimental, Instituto Nacional de Pediatría, Mexico City 04530, Mexico; jvelazqueza@pediatria.gob.mx; 9 Laboratorio de Neurociencias, Instituto Nacional de Pediatría, Secretaría de Salud, Mexico City 04530, Mexico; jmendozat@pediatria.gob.mx (J.G.M.-T.); ncardenasr@pediatria.gob.mx (N.C.-R.); 10 Laboratorio de Nutrición Experimental, Instituto Nacional de Pediatría, Secretaría de Salud, Mexico City 04530, Mexico; dortegac@pediatria.gob.mx; 11 Laboratorio de Neurociencia Traslacional, Escuela Superior de Medicina, Instituto Politécnico Nacional, Mexico City 11340, Mexico; crodriguezba@ipn.mx; 12 Red de Estudios Moleculares Avanzados, Clúster Científico y Tecnológico BioMimic^®^, Instituto de Ecología A.C. (INECOL), Xalapa 91073, Veracruz, Mexico; 13 Laboratorio de Bioquímica Genética, Instituto Nacional de Pediatría, Secretaría de Salud, Mexico City 04530, Mexico

**Keywords:** *Giardia lamblia*, 5-nitrothiazole, antigiardial activity, gene expression, molecular docking, molecular dynamics simulations

## Abstract

Giardiasis remains a significant global health issue, and its treatment is increasingly hampered by the emergence of drug resistance and cross-resistance between nitroimidazoles and nitazoxanide. In this study, we evaluated the in vitro antigiardial activity, cytotoxicity, and potential mechanisms of action of seven structural analogs (CLB 1–7) derived from a hybrid structure combining nitazoxanide and clofibric acid. Among them, compounds CLB-3 and CLB-5 emerged as the most potent candidates, demonstrating exceptional efficacy against a nitazoxanide-resistant (NTZr) strain of *Giardia lamblia*, with potencies up to 43 and 60 times higher than that of the reference drug, respectively. Furthermore, both compounds demonstrated high selectivity for the parasite, exhibiting low cytotoxicity in human Caco-2 cells. To investigate the mechanism of action, functional biochemical assays revealed that treatment with these derivatives significantly reduced the intracellular ATP levels and altered the ADP/ATP ratio, indicating a direct impact on the parasite’s energy metabolism. These functional findings correlate closely with RT-qPCR transcriptional analyses and in silico molecular dynamics simulations; collectively, the data suggest a multi-target mechanism of action that may involve the disruption of energy homeostasis and the possible inhibition of key enzymes in the glycolytic pathway (PFOR, G6PD::6PGL, PK, and PPDK). In summary, these results identify compounds CLB-3 and CLB-5 as highly promising chemotherapeutic candidates for combating refractory giardiasis.

## 1. Introduction

Giardiasis is a diarrheal infection caused by *Giardia lamblia* (*G. lamblia*), one of the most common human protozoal infections worldwide. The disease is particularly prevalent in developing countries, where an estimated 33% of the population has been affected. In developed countries, its prevalence is considerably lower, and most reported cases are associated with travel to regions where giardiasis is endemic [1]. Transmission occurs through ingestion of the cyst, the resistant form of *G. lamblia*, in contaminated water or food, as well as through direct person-to-person contact [2]. Many *G. lamblia* infections are asymptomatic and self-limited, which can complicate their detection. Symptomatic acute infections may present with abdominal pain, watery diarrhea, steatorrhea, and nausea. Chronic infection may cause recurrent diarrhea, malabsorption, weight loss, and vitamin deficiencies [3]. Timely and accurate diagnosis is therefore essential for effective treatment and the prevention of transmission. However, diagnosis remains challenging because the clinical manifestations are non-specific and may overlap with those of other gastrointestinal disorders [4].

The first-line pharmacological treatments include nitroimidazole derivatives such as metronidazole, tinidazole, and secnidazole, which disrupt essential metabolic and nucleic acid-related processes in the parasite. Benzimidazole drugs, including albendazole and mebendazole, act mainly by disrupting microtubule formation, thereby interfering with functions required for parasite survival [5,6,7]. When *G. lamblia* strains exhibit resistance to first-line nitroimidazoles and benzimidazoles, alternative treatments include nitazoxanide—which interferes with anaerobic energy metabolism—as well as furazolidone, quinacrine, chloroquine, or paromomycin. However, the clinical utility of nitazoxanide in these situations is increasingly compromised by the emergence of cross-resistance between nitroimidazoles and nitazoxanide [8,9]. This cross-resistance is primarily attributed to shared mechanisms of metabolic activation within the parasite, such as alterations in the pyruvate:ferredoxin oxidoreductase (PFOR) system and electron transport pathways [10]. Consequently, developing new chemical structures that can bypass these cross-resistance mechanisms is crucial. Despite the therapeutic options, treatment failure has been reported with increasing frequency, with failure rates reaching up to 45% for traditional nitroimidazoles after an initial course [11,12]. Therefore, the discovery and development of novel antigiardiasis drugs are important because of emerging drug resistance.

A highly attractive strategy for identifying new antigiardial agents involve the chemical modification of clinically used drugs to improve their biological activity [13,14,15]. In this regard, compounds containing a 5-nitrothiazole ring, such as nitazoxanide (NTZ), remain ideal pharmacophores because of their validated low-reduction potential, which allows them to serve as privileged electron acceptors during the parasite’s microaerophilic processes, including *G. lamblia* [16,17,18,19,20,21,22]. Concurrently, clofibric acid derivatives have gained interest in medicinal chemistry because of their lipophilic properties, can modulate cellular membrane permeability and restrict conformational flexibility when integrated into hybrid systems [23,24]. Because *G. lamblia* lacks conventional mitochondria and peroxisomes, it relies exclusively on a simplified, anaerobic central carbon metabolism—specifically through glycolysis and fermentative substrate-level phosphorylation—to generate ATP [8]. Consequently, the enzymes controlling these unique pathways have emerged as highly druggable targets.

This study describes the biological evaluation of a series of seven synthetic hybrid analogs of 5-nitrothiazole and clofibric acid (CLB 1–7) against *G. lamblia* trophozoites that are either susceptible (NTZs) or resistant (NTZr) to nitazoxanide. Using a multidisciplinary approach—combining in vitro phenotypic viability screening, cytotoxicity assessment in human intestinal Caco-2 cells, transcriptional profiling via RT-qPCR, and in silico ensemble docking coupled with 250 ns molecular dynamics simulations—we characterized the potency and biological target interaction of these derivatives. Our results identify compounds CLB-3 and CLB-5 as highly potent antigiardial candidates that trigger a multi-target disruptive mechanism, reducing the expression of key metabolic genes and compromising the parasite’s energy homeostasis, thereby establishing a promising chemical foundation for managing refractory giardiasis.

## 2. Results and Discussion

### 2.1. In Vitro Antigiardial Activity of Nitrothiazole-Clofibric Acid Analogs

**Figure 1:**
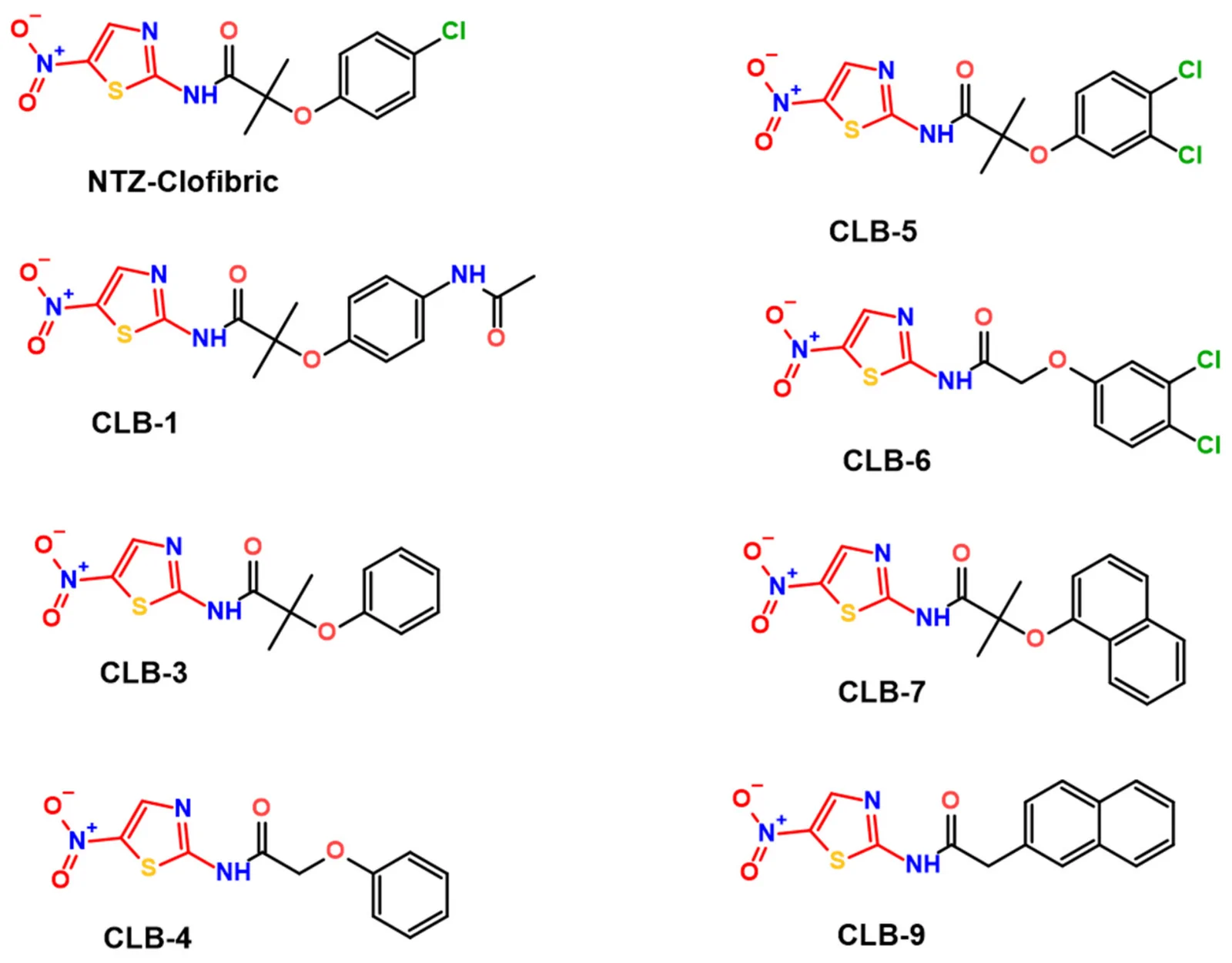
Chemical structures of the CLB compounds. The 5-nitrothiazole ring is shown in red (constitutes the common scaffold that bears the pharmacophore part), followed by the alkylamide substituents. The structures were prepared in ACD/ChemSketch (Freeware) 2020.2.0.

Our research group conducted a low-throughput screening (LTS) assay to identify novel synthetic compounds with activity against *G. lamblia* trophozoites. To this end, we screened an in-house library of 120 organic compounds synthesized at the Medicinal Chemistry Laboratory, Faculty of Pharmacy, Autonomous University of Morelos State. Among the compounds evaluated, we identified a series designated the CLB family, comprising seven molecules sharing a common 5-nitrothiazole ring as their key structural feature (Figure 1). The CLB compounds are nitrothiazole-clofibric acid chimeras, and their synthesis and chemical characterization have been previously reported [16,17,18,19,22]. The parental compound (NTZ-clofibric, Figure 1), which has shown significant activity against an NTZ-sensitive strain of *G. lamblia*, was 7-fold and 31-fold more active than nitazoxanide (NTZ) and metronidazole (MTZ), respectively [16]. Given the potency of the parent compound, we evaluated the seven analogs CLB-1, CLB-3, CLB-4, CLB-5, CLB-6, CLB-7, and CLB-9, against NTZ-sensitive and NTZ-resistant (NTZr) strains to determine whether the structural modifications retained or improved antigiardial activity.

**Figure 2:**
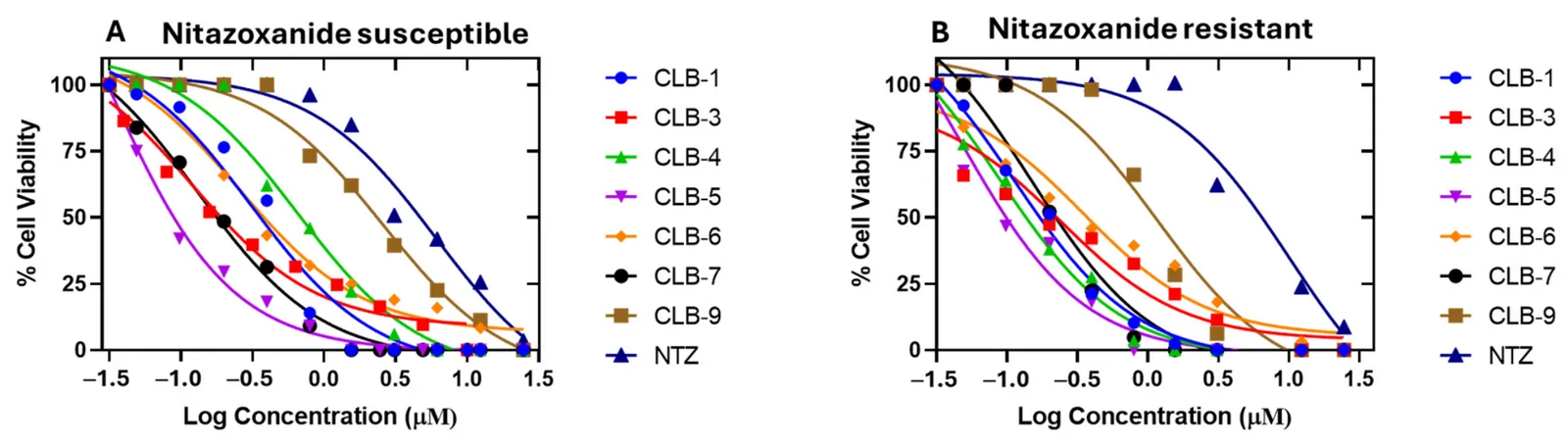
Effect of CLB compounds on the viability of *G. lamblia* trophozoites: (**A**) susceptible and (**B**) resistant strains. The trophozoites were incubated with increasing concentrations of each compound for 48 h at 37 °C, and trophozoite viability was subsequently determined. The values represent the mean ± SD of three independent experiments, each conducted with three replicates.

All tested analogs produced a concentration-dependent reduction in trophozoite viability in both strains (Figure 2A,B). Notably, the CLB derivatives were more active than NTZ under the experimental conditions used. In the NTZs strain, the IC_50_ values ranged from 0.07 to 1.70 µM, with CLB-5 being the most potent compound in the series, with an IC_50_ of 0.07 ± 0.004 µM, corresponding to approximately 60-fold greater activity than NTZ, which showed an IC_50_ of 4.22 ± 0.21 µM. CLB-3 was the second most active derivative, with an IC_50_ of 0.15 ± 0.008 µM, and was approximately 28-fold more potent than NTZ. CLB-6 also showed high activity, with an IC_50_ of 0.21 ± 0.001 µM. CLB-4 and CLB-7 displayed comparable potency, each with an IC_50_ of 0.28 ± 0.02 µM, and CLB-9 was the least active analog in the series, with an IC_50_ of 1.70 ± 0.10 µM. Nevertheless, it remained approximately 2.5-fold more active than NTZ (Table 1, Figure 2A).

When the compounds were evaluated against the NTZr strain, most CLB derivatives retained high antigiardial potency. CLB-1, CLB-3, and CLB-4 showed IC_50_ values of 0.36, 0.17, and 0.13 µM, respectively (Figure 2A), which were comparable to or lower than those obtained in the NTZ-sensitive strain. These compounds were approximately 20-, 43-, and 56-fold more potent, respectively, than NTZ, which exhibited an IC_50_ of 7.24 ± 0.40 µM against the NTZr strain. CLB-5 remained the most active derivative against the resistant strain, with an IC_50_ of 0.12 ± 0.006 µM, corresponding to approximately 60-fold greater potency than NTZ (Figure 2B). CLB-7, CLB-6, and CLB-9 showed IC_50_ values of 0.38 ± 0.02 µM, 0.56 ± 0.03 µM, and 1.07 ± 0.05 µM, respectively, and were 19-, 13-, and 7-fold more active than NTZ (Table 1). Thus, although some variation in potency was observed between the NTZs and NTZr strains, all tested derivatives remained more active than NTZ against the resistant phenotype. The results of the antigiardial activity demonstrate that CLB-5 was the most potent compound of the series in both the susceptible (IC_50_: 0.07 μM) and resistant (IC_50_: 0.12 μM) strains, showing 60 times greater activity than NTZ in both strains; although CLB-4 showed good activity in the resistant strain with an IC_50_ of 0.13 μM and a potency of 56, in the susceptible strain, its IC_50_ was 0.28 μM and a potency of 15, therefore CLB-4 was not selected for subsequent studies.

Interestingly, CLB-5 and NTZ displayed an identical resistance index (RI = 1.71). This comparable profile suggests that the resistance mechanisms established in the NTZr strain exert a similar relative impact on both chemical compounds. Nevertheless, CLB-5 represents a major improvement because its absolute inhibitory concentration against the resistant strain (IC50 = 0.12 μM) remains orders of magnitude lower than the concentration required by NTZ to inhibit even the sensitive strain (IC50 = 4.22 μM). Furthermore, an RI below 2 indicates that CLB-5 preserves high susceptibility and effectively circumvents high-level resistance [25].

### 2.2. Chemical Structure Analysis and Antigiardial Activity

The preservation of activity in the NTZr strain suggests that CLB derivatives may be less affected by the cellular alterations associated with NTZ resistance. However, these findings do not establish that the compounds directly overcome a specific mechanism of resistance. To explore possible relationships between chemical structure and antigiardial activity, the principal structural features of the CLB series were compared. All derivatives retained the 5-nitrothiazol ring, a nitroheterocyclic moiety also present in NTZ. This group is considered important for biological activity because it can serve as an electron acceptor during reductive metabolic processes in the parasite [26,27]. Nevertheless, the differences in potency observed across the series indicate that the remaining structural features also influence activity.

CLB-1, CLB-3, CLB-5, and CLB-7 are propanamide derivatives, containing two methyl groups adjacent to the amide moiety (-C(CH_3_)_2_-). This branch may increase lipophilicity and restrict conformational flexibility and rigidity [28,29]. Notably, CLB-5, the most potent compound in the series, contains this structural feature. In contrast, CLB-4, CLB-6, and CLB-9 are acetamide derivatives and contain methyl groups (-CH_2_-) at the corresponding position. The influence of this substitution is particularly evident when CLB-5 and CLB-6 are compared. Both compounds contain a 3,5-dichlorophenoxy group, but CLB-5 possesses a dimethyl-substituted propanamide linker, whereas CLB-6 contains a less substituted acetamide linker. CLB-5 was approximately 5-fold more potent than CLB-6 in the NTZr strain, suggesting that the two methyl groups contribute favorably to activity. This effect may arise from changes in lipophilicity, molecular conformation, membrane permeability, or interactions with a biological target, although these possibilities require experimental confirmation. Substitution of the terminal aromatic ring also appeared to affect potency. CLB-5 and CLB-6 contain a 3,5-dichlorophenoxy group and showed greater activity than their corresponding analogs with the unsubstituted aromatic rings CLB-3 and CLB-4. Chlorine substitution can increase lipophilicity and alter the electronic properties of aromatic systems through inductive electron-withdrawing effects [30,31]. These changes may improve membrane passage or favor interactions with parasite biomolecules. However, because several physicochemical properties are modified simultaneously, the contribution of chlorine substitution cannot be attributed to a single factor based solely on the present data. CLB-7 and CLB-9 contain a larger naphthalene moiety, which may introduce additional steric constraints. Furthermore, unlike the other derivatives, CLB-9 lacks an ether linkage between the aromatic system and the aliphatic chain; instead, the naphthalene group is connected directly to the methylene group. This modification may alter the orientation and conformational flexibility of the molecule and could partly explain its lower potency relative to most members of the series. Overall, the structure–activity analysis suggests that the dimethyl-substituted propenamide linker and the 3,5-dichlorophenoxy group favor antigiardial potency, whereas increased steric bulk and removal of the ether linkage may be less favorable. Based on their potency against both NTZs and NTZr strains, CLB-3, CLB-5, CLB-6, and CLB-7 were selected for subsequent studies.

### 2.3. Cytotoxicity in Caco-2 Cells and Selectivity Index (SI)

**Figure 3:**
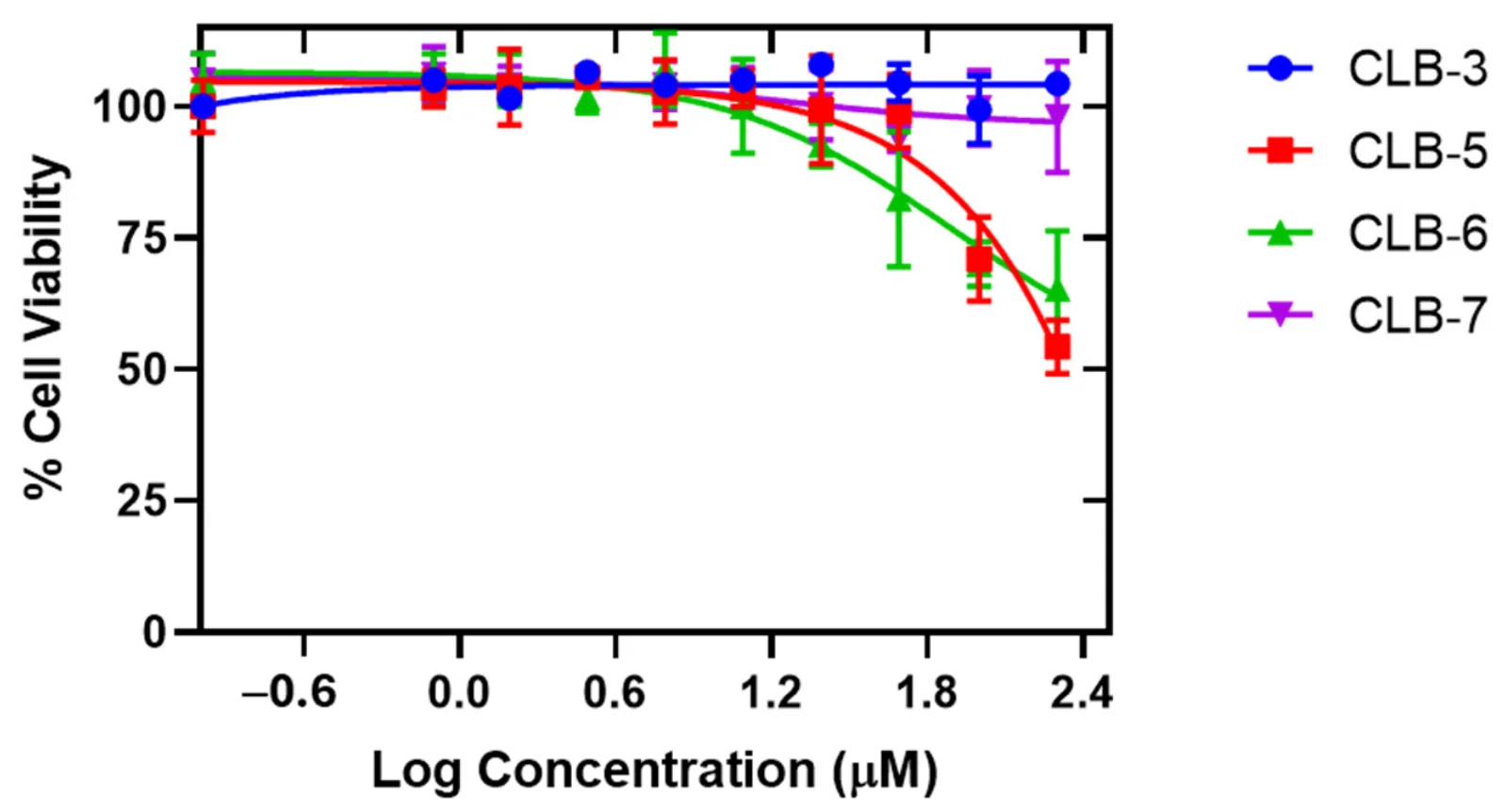
Cytotoxic effect of CLB compounds on Caco-2 cells. The Caco-2 cells were used to generate concentration–response curves to determine cell viability. The cells were treated with compounds CLB-3, CLB-5, CLB-6, and CLB-7, and cell viability was determined using the XTT cell proliferation assay. The values represent the mean ± standard deviation from three independent experiments each conducted with three replicates.

To evaluate the safety profile of the most promising compounds, we assessed the cytotoxicity of CLB-3, CLB-5, CLB-6, and CLB-7 in Caco-2 cells, an in vitro model of the intestinal epithelium. CLB-3 and CLB-7 exhibited no significant cytotoxicity over the concentration range tested, maintaining cell viability close to 100% even at the highest concentrations. In contrast, CLB-5 and CLB-6 reduced cell viability only at the highest concentration evaluated, with viability decreasing to approximately 50–65% at 500 µM. These findings contrast with the high potency of the compounds, which exhibited IC_50_ values ranging from 0.12 to 0.38 µM against *G. lamblia*. In the Caco-2 cells, viability decreased only at concentrations near 100–250 µM (Figure 3). This marked difference between antigiardial activity and cytotoxicity resulted in selectivity index (SI) values greater than those obtained for NTZ in both strains (Table 1). The high SI values indicate that the CLB derivatives were considerably more active against *G. lamblia* trophozoites than against Caco-2 cells under the experimental conditions used. These results suggest favorable preliminary selective profiles. Selectivity is an important parameter in the early evaluation of drug candidates because insufficient discrimination between parasite and host cells may increase the risk, toxicity [32]. In general, a high SI reflects a substantial difference between cytotoxic and antiparasitic concentrations. An SI value of ≥10 has been proposed as a useful threshold for selecting compounds for further investigation [33], whereas other authors have suggested that values above 20 may indicate favorable selectivity [34]. All four derivatives tested in this study exhibited SI values greater than 100. Among them, CLB-3 and CLB-7 showed the most favorable cytotoxicity profiles, as cell viability remained largely unaffected across the tested concentration range. Taken together, these results support the further evaluation of CLB-3, CLB-5, CLB-6 and CLB-7 as potential antigiardial candidates.

### 2.4. Gene Expression Profiles of Giardia lamblia Trophozoites Exposed to CLB-3 and CLB-5

#### 2.4.1. Transcriptional Alterations in Glycolytic and Fermentative Pathway Genes

To investigate transcriptional response to compound exposure, we evaluated the effect of CLB-3 and CLB-5 on 13 genes involved in energy metabolism, redox homeostasis, and cytoskeletal organization in both the NTZs and NTZr strains of *G. lamblia* trophozoites. The results revealed distinct transcriptional profiles that depended on both the compound and the strain tested.

**Figure 4:**
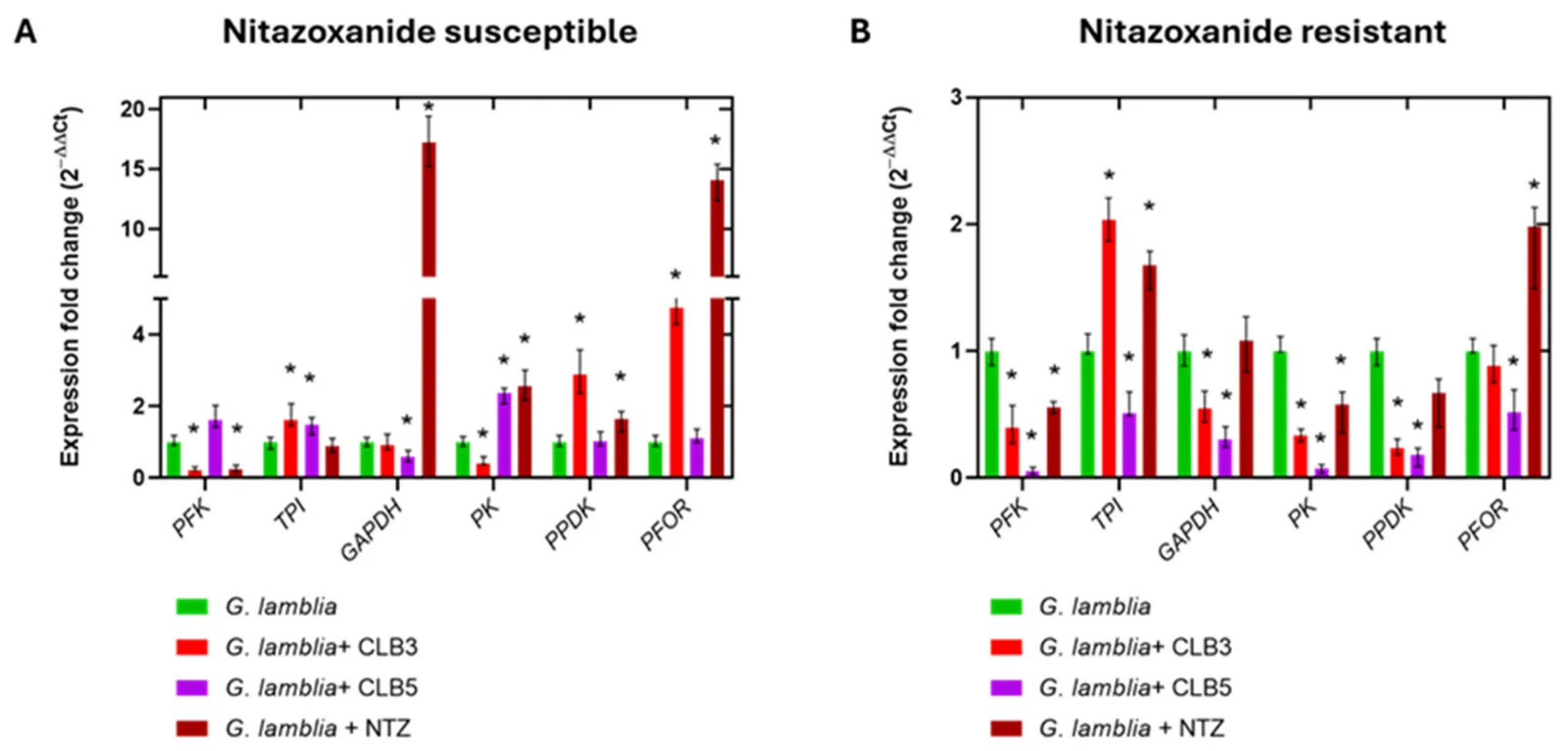
Differential expression of glycolysis—and fermentation-associated genes in *G. lamblia* trophozoites treated with the compounds CLB-3, CLB-5, and nitazoxanide (NTZ). (**A**) NTZ-sensitive (NTZs) strain. (**B**) NTZ-resistant (NTZr) strain. Transcript levels of phosphofructokinase (*PFK*), triosephosphate isomerase (*TPI*), glyceraldehyde-3-phosphate dehydrogenase (*GAPDH*), pyruvate kinase (*PK*), pyrophosphate-dependent pyruvate phosphate dikinase (*PPDK*), and pyruvate-ferredoxin oxidoreductase (*PFOR*) were determined by RT-qPRT. Changes in expression were determined by RT-qPCR and calculated using the 2^−ΔΔCt^ method with *ALDO* as the reference gene. Data represent the mean ± SD (n = 3 biological replicates). The asterisk (*) indicates a significant difference (*p* < 0.05 vs. untreated trophozoites of the corresponding strain) in expression based on the Mann–Whitney test.

In the NTZs strain, CLB-3 markedly reduced the transcript levels of phosphofructokinase (*PFK*), and pyruvate kinase (*PK*) by 9-, and 2.5-fold, respectively, whereas glyceraldehyde-3-phosphate dehydrogenase (*GAPDH*) expression did not differ significantly from that of the untreated trophozoites (Figure 4A). In contrast, triosephosphate isomerase (*TPI*), pyrophosphate-dependent pyruvate phosphate dikinase (*PPDK*), and pyruvate oxidoreductase (*PFOR*) were upregulated by 1.6-, 2.9-, and 4.7-fold, respectively. CLB-5 produced a less pronounced transcriptional response in the NTZs strain. *PPDK* and *PFOR* transcript levels remained similar to those in the untreated control, whereas *PFK*, *TPI*, and *PK* were moderately upregulated by 1.6-, 1.4-, and 2.3-fold, respectively. *GAPDH* expression decreased 1.7-fold. NTZ decreased the *PFK* transcript levels by 4-fold, while *TPI* expression remained unchanged. In contrast, *GAPDH*, *PK*, *PPDK*, and *PFOR* were upregulated by 17.2-, 2.5-, 1.6-, and 14.1-fold, respectively (Figure 4A). These differences indicate that CLB-3 and CLB-5 do not reproduce the transcriptional response elicited by NTZ in the NTZs strain.

A substantially different response was observed in the NTZr strain. CLB-3 increased *TPI* expression 2-fold, a response similar in direction to that induced by NTZ, which produced a 1.7-fold increase (Figure 4B). However, CLB-3 reduced the *PFK*, *GAPDH*, *PK*, and *PPDK* transcript levels by 2.5-, 2-, 3.3-, and 4.3-fold, respectively. In comparison, NTZ reduced the expression of *PFK*, *PK*, and *PPDK* by 2-, 1.7-, and 1.5-fold, respectively. CLB-5 induced the broadest repressive response in the resistant strain. Transcript levels of *PFK*, *TPI*, *GAPDH*, *PK*, *PPDK*, and *PFOR* decreased by 20-, 2-, 3.3-, 14-, 5.5-, and 2-fold, respectively (Figure 4B). Thus, unlike the more limited or mixed responses observed with CLB-3 and NTZ, CLB-5 consistently downregulated all glycolytic and fermentative genes examined in the NTZr strain.

Overall, CLB-3 and CLB-5 produced compound- and strain-dependent changes in the transcription of genes associated with the central carbon metabolism. The widespread downregulation induced by CLB-5 in the NTZr strain was particularly notable because it coincided with the high antigiardial potency of this derivative and indicates a significant change in energy metabolism, which is relevant, as glycolytic proteins have been proposed and characterized as potential drug targets for *G. lamblia* [35,36]. Energy metabolism is essential for *G. lamblia* survival because the parasite lacks conventional mitochondria and relies primarily on substrate-level phosphorylation through glycolytic and fermentative pathways to generate ATP [37]. Enzymes involved in these pathways have therefore been proposed as potential pharmacological targets [35,36]. The reduction in *PFK*, *PK*, *PPDK*, and *PFOR* transcript levels may compromise several steps involved in carbon flux, pyruvate metabolism, and ATP production, particularly in the NTZr- strain. The decrease in PPDK expression is also relevant, as this enzyme contributes to an alternative ATP-generating reaction in *G. lamblia*. Its simultaneous downregulation with PFK and PK suggests that CLB-5 may limit the transcriptional capacity of more than one route involved in energy production. However, measurements of enzyme activity, protein and metabolite abundance, and glucose consumption will be required to determine whether these transcriptional changes result in functional metabolic impairment. Several glycolytic enzymes in *G. lamblia* differ substantially from their mammalian counterparts, and in some cases, show greater similarity to bacterial enzymes [38]. These differences support the investigation of parasite energy metabolism as a source of selective therapeutic targets.

#### 2.4.2. Effect on Pentose Phosphate Pathway Genes and Redox Response

**Figure 5:**
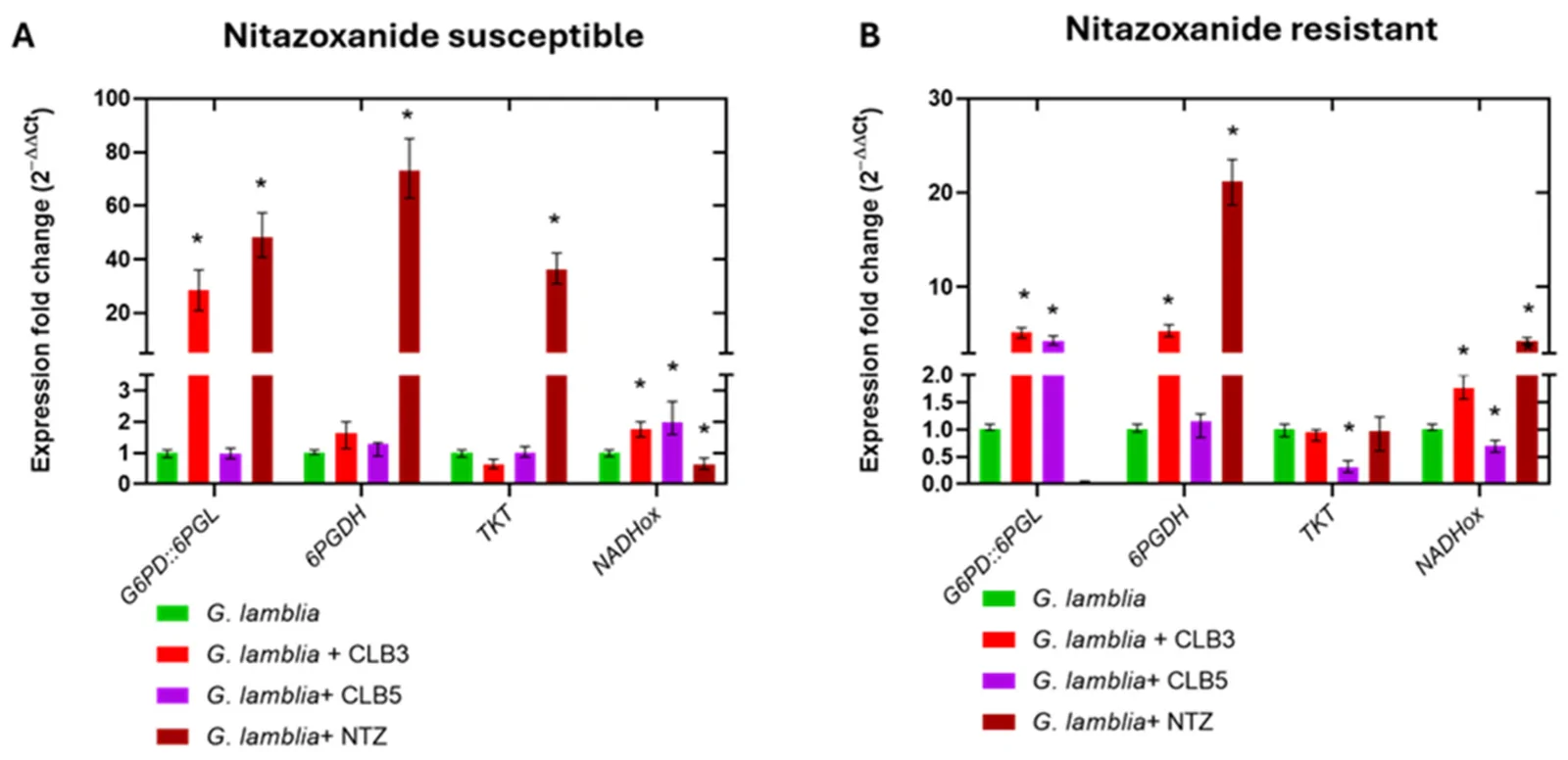
Differential expression of the pentose phosphate pathway- and redox-associated genes in *G. lamblia* trophozoites treated with the compounds CLB-3, CLB-5, or nitazoxanide (NTZ). (**A**) NTZ-sensitive strain (NTZs), (**B**) NTZ-resistant (NTZr) strain. Transcript levels of glucose-6-phosphate dehydrogenase::6-phosphogluconolactone (*G6PD*::*6PGL*), 6-phosphogluconate dehydrogenase (*6PGDH*), transketolase (*TKT*), and NADH-oxidase (*NADH*ox) were determined by RT-qPCR. Changes in expression were determined by RT-qPCR and calculated using the 2^−ΔΔCt^ method with *ALDO* as the reference gene. Data represent the mean ± SD (n = 3 biological replicates). The asterisk (*) indicates a significant difference (*p* < 0.05 vs. untreated trophozoites of the corresponding strain) in expression based on the Mann–Whitney test.

Analysis of genes associated with the pentose phosphate pathway (PPP) revealed distinct compound- and strain-dependent transcriptional responses. In the NTZs strain, CLB-3 increased the expression of *G6PD::6PGL* and *6PGDH* by 28-, and 1.6-fold, respectively. A similar but more pronounced response was observed after NTZ exposure, which increased the *G6PD::6PGL*, *6PGDH*, and transketolase (*TKT*) transcript levels by 48-, 73-, and 36-fold, respectively. *G6PD::6PGL* and *6PGDH* encode enzymes involved in the oxidative phase of PPP, which contributes to NADPH production [39,40]. The marked induction of these genes by CLB-3 and NTZ in the NTZs strain is consistent with activation of a compensatory redox response. In contrast, CLB-5 induced only limited changes in PPP-associated transcripts in the NTZs strain, with expression levels remaining close to those of the untreated trophozoite controls (Figure 5A). This relatively modest response differs from the broader transcriptional effects observed in glycolytic genes and suggests that in the sensitive strain, CLB-5 may affect central carbon metabolism without strongly activating the PPP at the transcriptional levels.

A different expression pattern was observed in the NTZr strain. CLB-3 increased the *G6PD::6PGDH*, and *6PGDH* transcript levels by 5.2-fold each, whereas *TKT* expression remained unchanged. NTZ selectively increased *6PGDH* expression by 21.2-fold but reduced the *G6PD::6PGL* transcript levels by 25-fold. CLB-5 produced a mixed response in the resistant strain. *G6PD::6PGL* expression increased by 2.4-fold, whereas the *TKT* and *NADH oxidase* (*NADHox*) transcript levels decreased by 2.9- and 2.4-fold, respectively (Figure 5B). This pattern suggests that CLB-5 does not uniformly repress all redox-associated genes. Instead, it appears to induce a selective transcriptional imbalance within pathways involved in NADPH generation, carbon distribution, and redox homeostasis.

The coordinated induction of *G6PD::6PGL*, and *6PGDH* by CLB-3, particularly in the TNZs strain, may reflect increased demand for NADPH. The oxidative branch of the PPP is an important source of reducing equivalents in *G. lamblia* and contributes to antioxidant defense, biosynthetic processes, and the maintenance of cellular homeostasis under microaerophilic conditions [41,42,43]. Accordingly, the expression patterns observed after CLB-3 and NTZ are compatible with activation of a stress-responsive metabolic program. The different responses observed between NTZs and NTZr trophozoites further indicate that resistance is associated with altered regulation of carbohydrate metabolism and redox-related pathways. In particular, the opposite effects of NTZ on *G6PD::6PGL* expression in the two strains suggest that the resistant phenotype may modify the way trophozoites redistribute metabolic flux or regulate NADPH-generating enzymes after drug exposure.

NADHox contributes to redox balance in *G. lamblia* by oxidizing NADH and reduced molecular oxygen, thereby helping maintain the intracellular NAD^+^/NADH ratio and limiting oxygen-associated stress [44,45]. In the NTZs strain, both CLB-3 and CLB-5 increased the expression of *NADHox*; these responses are consistent with the activation of compensatory mechanisms involved in redox homeostasis.

In contrast, the reduction in *NADox* expression induced by CLB-5 in the NTZr strain may restrict the parasite’s capacity to maintain electron balance under compound exposure. Together with the repression of *TKT* and the broad downregulation of glycolytic genes described above, this result suggests that CLB-5 disrupts several transcriptional components of energy and redox metabolism in resistant trophozoites.

#### 2.4.3. Transcriptional Effects on Cytoskeletal and Virulence-Associated Genes

**Figure 6:**
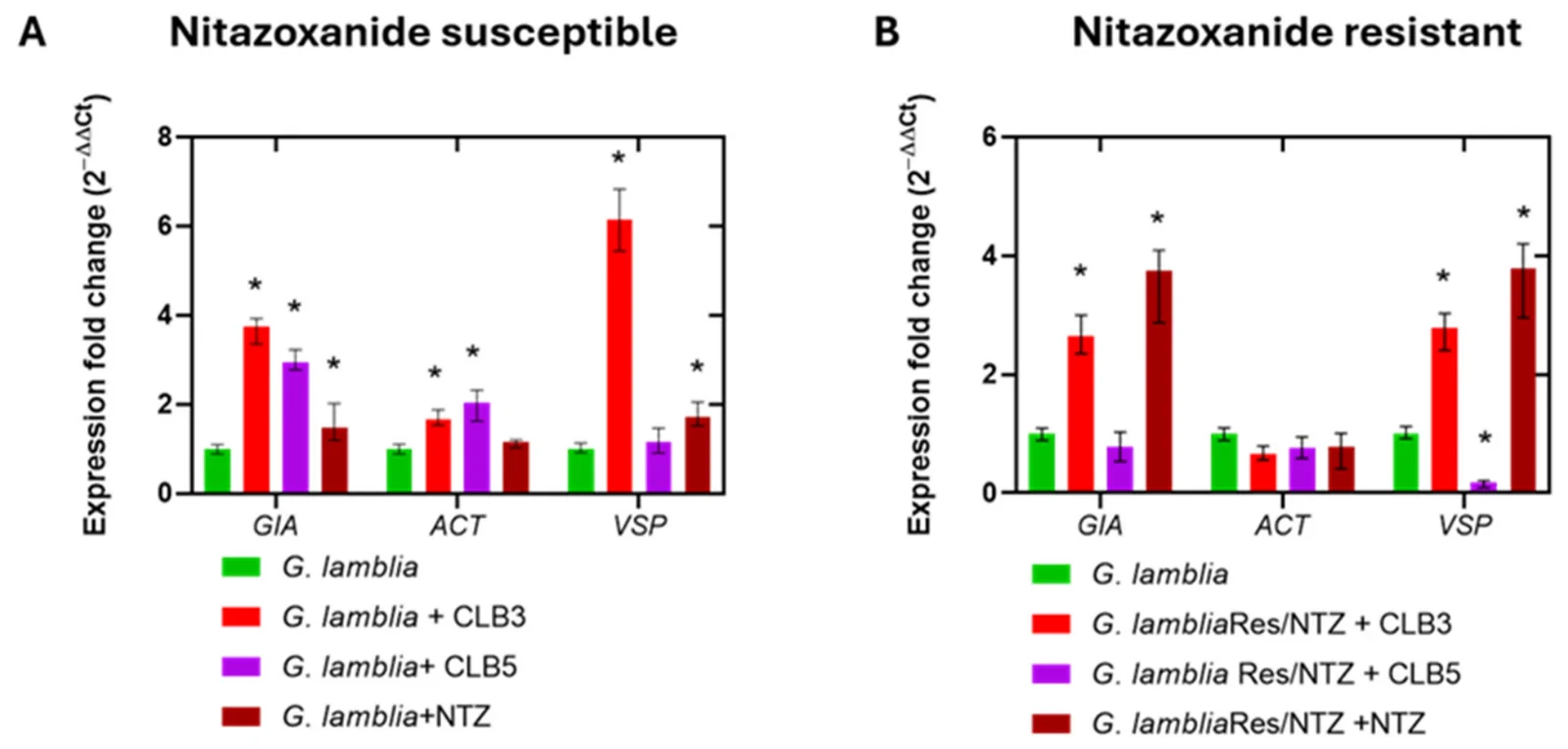
Differential expressions of giardin (*GIA*), actin (*ACT*), and variant-specific surface proteins (*VSP*) in *G. lamblia* trophozoites exposed to CLB-3, CLB-5, and nitazoxanide (NTZ). (**A**) NTZ-sensitive (NTZs) strain; (**B**) *G.* NTZ-resistant (NTZr) strain. Changes in expression were determined by RT-qPCR and calculated using the 2^−ΔΔCt^ method with *ALDO* as the reference gene. Data represent the mean ± SD (n = 3 biological replicates). The asterisk (*) indicates a significant difference (*p* < 0.05 vs. untreated trophozoites of the corresponding strain) in expression based on the Mann–Whitney test.

The expression profiles of genes associated with cytoskeleton and cell surface components (giardin: *GIA*, actin: *ACT*, and variant-specific surface protein: *VSP*) showed marked differences depending on both the compound and the strain analyzed. In the NTZs strain, CLB-3 promoted *GIA* and *VSP* transcript levels by 3.7- and 2.6-fold, respectively (Figure 6A). CLB-5 also induced *GIA* expression by 2.9-fold and increased the *ACT* transcript levels 2-fold. In contrast, NTZ produced only a modest 1.4-fold increase in *GIA* expression, which was not significantly different from that observed in the untreated trophozoites (Figure 6A).

A distinct transcriptional pattern was observed in the NTZr strain; CLB-3 and NTZ induced marked upregulation of *GIA* and *VSP* (Figure 6B). In contrast, CLB-5 strongly reduced the *VSP* transcript levels, whereas *GIA* and *ACT* expression remained similar to that of the untreated controls. The pronounced reduction in *VSP* expression after CLB-5 exposure may indicate the disruption of processes involved in surface remodeling or antigenic variation. Giardins are structural proteins unique to *G. lamblia*, and are associated with the ventral disc, flagella, and other cytoskeletal structures. They contribute to cell shape, attachment, and motility [46]. Actin also participates in essential processes such as cytokinesis, membrane trafficking, and the maintenance of cellular architecture. Therefore, changes in *GIA* and *ACT* expression may reflect a compensatory response to structural stress induced by compound exposure.

Overall, these results indicate that CLB-3 and CLB-5 induce distinct transcriptional responses in genes related to cytoskeletal organization and the parasite surface. CLB-3 primarily promoted gene upregulation, whereas CLB-5 produced a more selective response characterized by the repression of *VSP* in the resistant strain. This difference further supports the possibility that the two compounds affect *G. lamblia* through partially distinct cellular responses.

### 2.5. Effects of CLB-3 and CLB-5 on Intracellular ATP and ADP Levels

**Figure 7:**
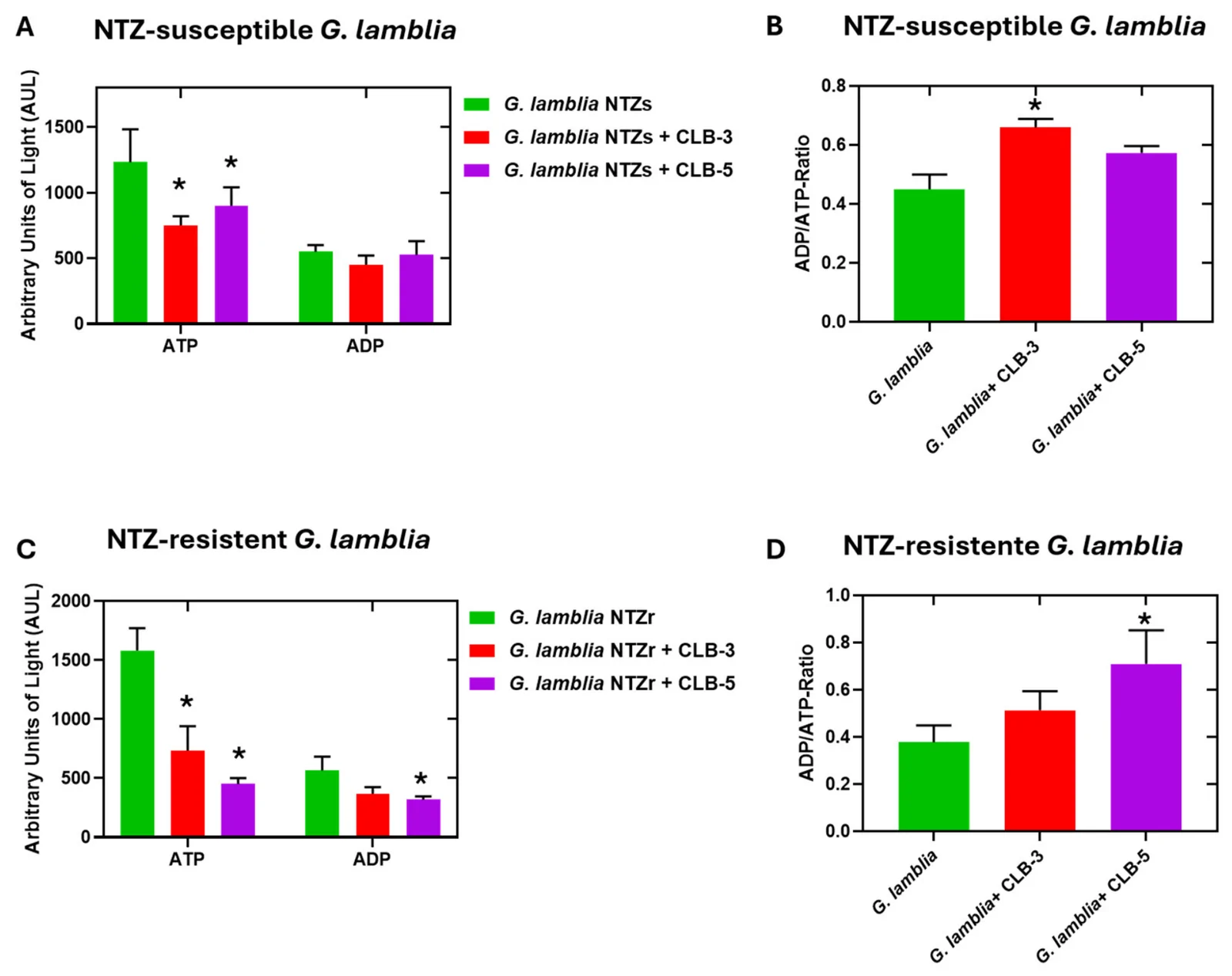
Effect of CLB-3 and CLB-5 on ATP and ADP levels and the ADP/ATP ratio in NTZ-susceptible and -resistant *G. lamblia* strains. (**A**) Intracellular ATP and ADP levels, and (**B**) quantitative ADP/ATP ratio in NTZ-susceptible (NTZs) *G. lamblia* trophozoites. (**C**) ATP and ADP levels, (**D**) ADP/ATP ratio in NTZ-resistant (NTZr) *G. lamblia* trophozoites. Values represent the mean ± standard deviation (SD) of three independent experiments performed with three replicates each. Statistical analysis was performed using the non-parametric Mann–Whitney test. Asterisks (*) indicate a statistically significant difference *p* < 0.05 compared to the untreated trophozoites of the corresponding strain.

To determine whether the transcriptional changes induced by CLB-3 and CLB-5 translate into functional metabolic alterations, intracellular ATP and ADP levels and ADP/ATP ratio were quantified in both the NTZ-susceptible and NTZ-resistant strains. The results revealed changes in the energy status of *G. lamblia* trophozoites exposed to CLB-3 and CLB-5. In the NTZs strain, CLB-3 compounds reduced the ATP and ADP levels compared to the control, although the increase in the ADP/ATP ratio indicates a shift in the energy balance toward a state of lower ATP availability; on the other hand, CLB-5 induced a decrease in ATP levels; however, the ADP/ATP ratio showed no significant differences compared to the control (Figure 7A,B). The effect was more pronounced in the NTZr strain, particularly following treatment with CLB-5, which caused the greatest decrease in ATP and a marked increase in the ADP/ATP ratio, whereas CLB-3 showed an intermediate effect (Figure 7C,D). Taken together, these changes suggest that both compounds disrupt energy nucleotide homeostasis, with CLB-5 causing the most significant energy perturbation in the NTZr strain. The changes observed in ADP and ATP levels are consistent with the transcriptional alterations identified in genes associated with energy metabolism. The coordinated decrease in these genes suggests the reduced capacity of glycolysis and fermentative pathways to sustain ATP production, which could explain the observed ATP/ADP imbalance. In contrast, CLB-3 produced a less pronounced energy disturbance, supporting the hypothesis that disruption of central carbon metabolism constitutes a relevant component of its antigiardial activity.

### 2.6. Molecular Simulations

#### 2.6.1. Ensemble Docking Predicts More Favorable Binding of CLB-3 and CLB-5 than NTZ to Selected *Giardia lamblia* Proteins

**Figure 8:**
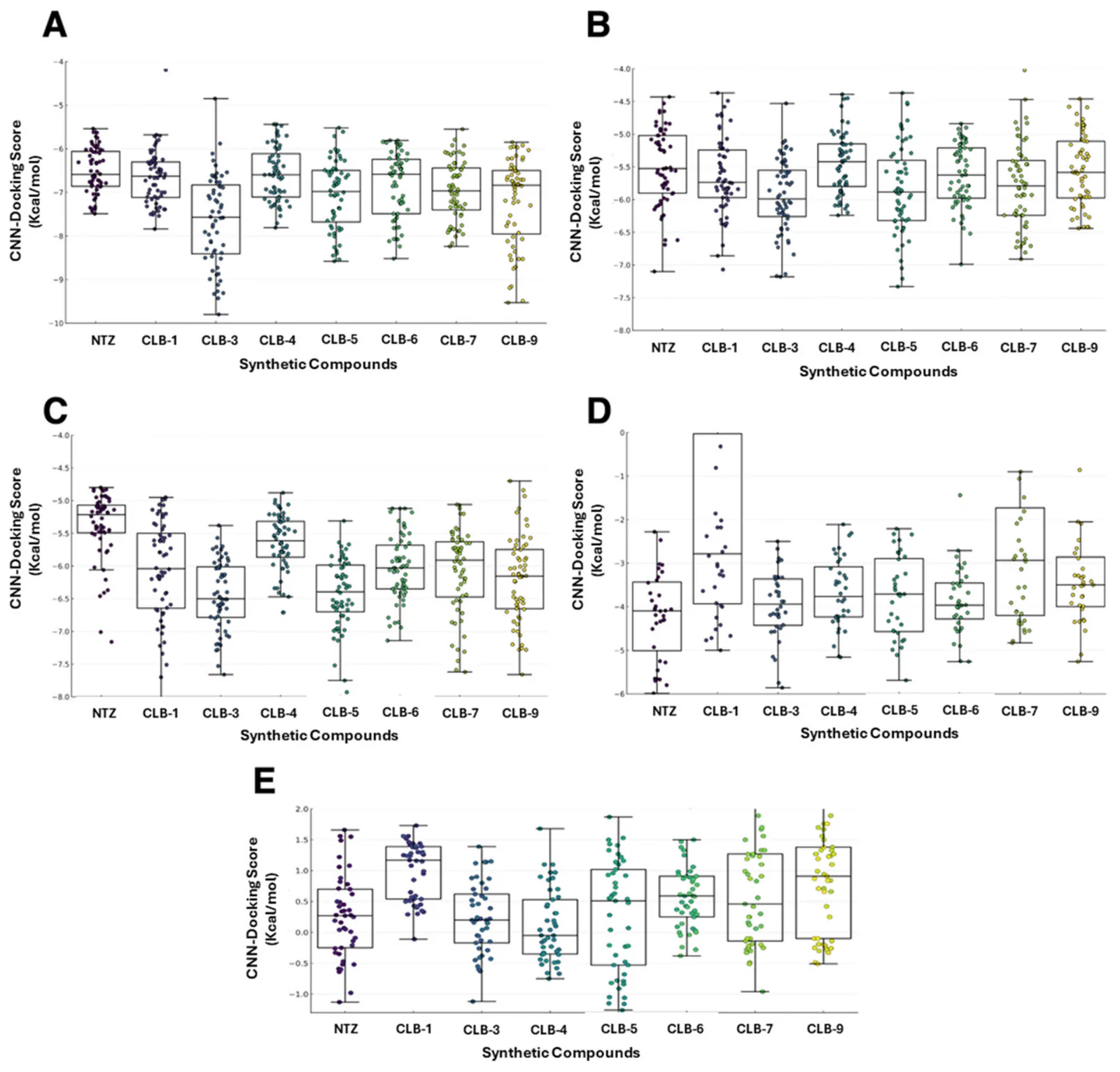
Comparative molecular docking scores of nitazoxanide (NTZ) and CLB derivatives within essential metabolic enzymes of *G. lamblia*. Calculated docking scores showing NTZ (control) and CLBs within the catalytic pockets of (**A**) G6PD::6PGL, (**B**) PK, (**C**) PPDK, (**D**) PFOR, and (**E**) giardin. Each point represents the estimated binding energy (kcal/mol) for a specific conformational position. Box plots show the median (center line), interquartile range (box), and maximum and minimum non-outlier values (whiskers). More negative values indicate a higher theoretical binding affinity.

To explore potential molecular interactions underlying the antigiardial activity of the CLB series, we performed ensemble docking against five proteins associated with essential metabolic or structural processes in *G. lamblia*: G6PD::6PGL, PK, PPDK, PFOR, and GIA. Docking distributions for the CLB derivatives were compared with those obtained for NTZ across the protein conformations included in the ensembles (Figure 8). Among the compounds evaluated against G6PD::6PGL, CLB-3 showed the most favorable docking scores, with values ranging from approximately −9.2 to −8.6 kcal/mol (Figure 8A). These values were more favorable than those obtained for NTZ, suggesting that CLB-3 may establish stable interactions within the predicted binding region of this enzyme. Because G6PD::6PGL has been proposed as a molecular target of NTZ [47], the predicted interaction with CLB-3 supports further investigations of this derivative as a potential inhibitor of this enzyme. CLB-3 and CLB-5 also showed favorable predicted binding to PK and PPDK. Their best docking scores reached approximately −6.9 and −6 kcal/mol, respectively, compared with −5.5 and −5 kcal/mol for NTZ (Figure 8B,C). These results suggest that the two derivatives may interact more favorably than NTZ with the binding regions identified in these enzymes. This observation is particularly relevant because PK and PPDK participate in ATP-generating reactions that are essential to the energy metabolism of *G. lamblia*. For PFOR, the docking-score distributions showed less discrimination among the tested ligands. NTZ and most CLB derivatives displayed broadly comparable values, whereas CLB-1 and CLB-7 showed less favorable predicted interactions (Figure 8D). PFOR plays a central role in anaerobic pyruvate metabolism, and nitroheterocyclic drugs have been associated with interference in PFOR-linked metabolism processes [47,48,49]. Because the CLB family possesses the nitrotiazole moiety present in the NTZ, their interaction with PFOR remains plausible. However, the similar docking profiles observed across most of the series do not identify PFOR as a selective target of CLB-3 and CLB-5. Docking against giardin yielded unfavorable or near-zero for most compounds (Figure 8E), suggesting limited complementarity with the evaluated binding regions. These results indicate that none of the compounds bind well to giardin, so they are probably not part of its mechanism of action. Overall, CLB-3 and CLB-5 showed the most favorable docking profiles across several of the proteins evaluated, particularly G6PD::6PGL, PK and PPDK, and their score distributions were comparable to or more favorable than those of NTZ for four out of the five targets (Table S1). These findings are consistent with the possibility that the derivatives interact with multiple proteins involved in parasite energy metabolism rather than acting through a single exclusive target.

Analysis of the predicted binding poses revealed key hydrogen-bonding and electrostatic interactions that may contribute to ligand recognition. CLB-3 showed particularly favorable poses within the predicted binding regions of G6PD::6PGL and PFOR, whereas CLB-5 interacted with catalytically relevant regions of PK and PPDK. These findings suggest that members of the CLB series may engage in diverse enzymes related to glycolysis. In the predicted G6PD::6PGL-CLB-3 complex, the derivative adopted a deeper, more energetically favorable orientation within the NADP^+^-dependent catalytic pocket and extended toward the ribose-phosphate binding region. In contrast, NTZ was positioned closer to the peripheral region of the cofactor site. This difference in orientation may allow CLB-3 to establish a broader interaction network within the pocket. CLB-3 was also predicted to bind near the TPP and CoA-associated channel of PFOR. In contrast, NTZ remained closer to the entrance of the modeled cavity. The location of the CLB-3 pose suggests that the derivative could interfere with substrate or cofactor access.

Within PPDK, CLB-5 occupied a predicted binding region in the nucleotide-dependent phosphotransferase domain. In PK, CLB-5 was positioned near the ATP/Mg^2+^-associated catalytic cleft and formed polar interactions with residues involved in ligand recognition. These poses are consistent with possible interactions at functionally relevant regions of both enzymes.

Overall, CLB-3 and CLB-5 were predicted to occupy catalytically relevant regions of all four enzymes more consistently than NTZ. These results support a multitarget hypothesis in which the CLB derivatives may affect several components of *G. lamblia* energy metabolism. Taken together, these findings support further dynamic investigation of CLB-3 and CLB-5.

#### 2.6.2. Molecular Dynamics Simulations Reveal Stable Binding Profiles for CLB-3 and CLB-5

**Figure 9:**
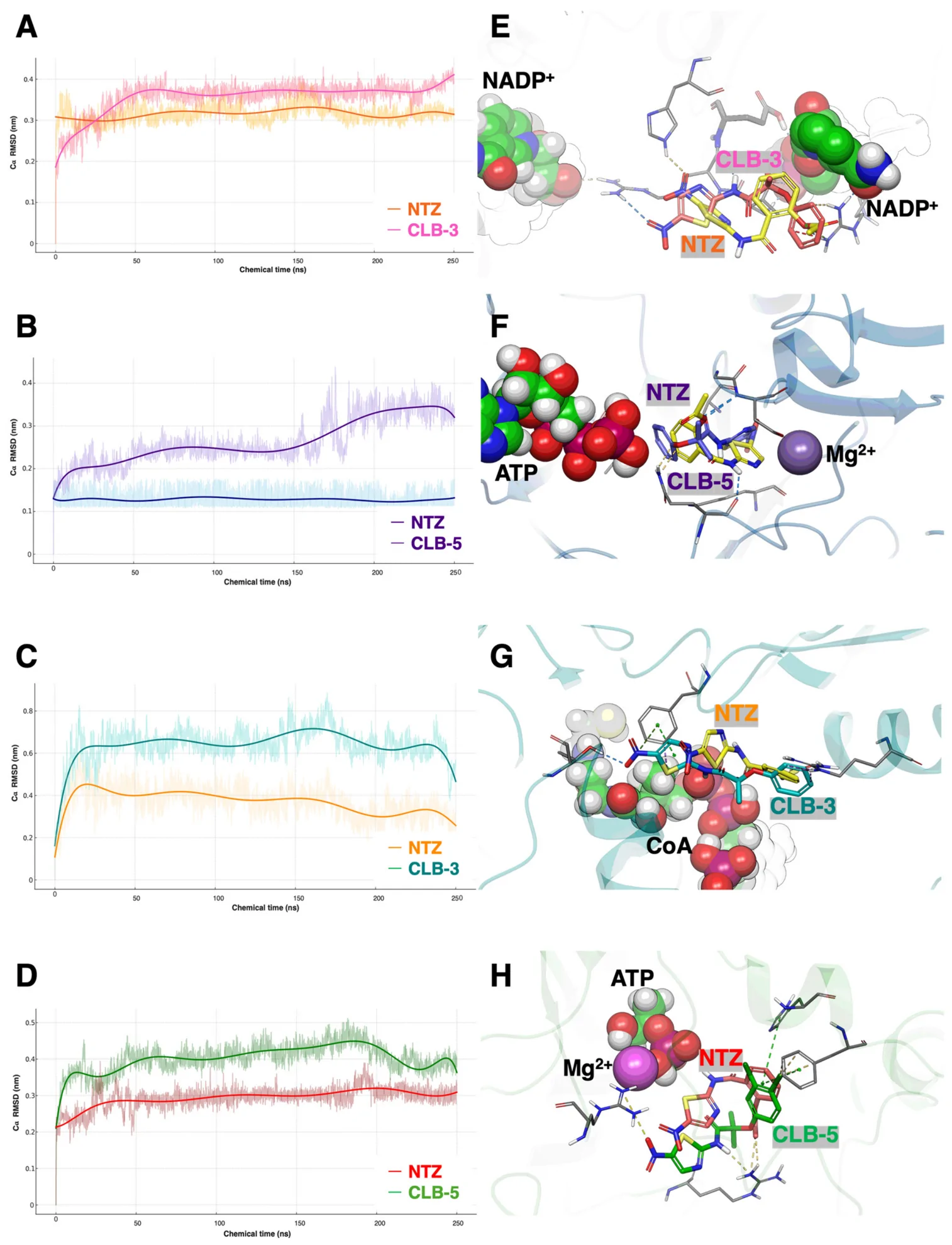
Molecular docking analysis and dynamics simulations of CLB-3 and CLB-5 complexed to key enzymes of *G. lamblia* metabolism. The panels (**A**–**D**) show the root mean square deviation (RMSD) traces of the alpha carbons (Cα RMSD) over 250 ns of simulation for the G6PD:6PGL, PK, PFOR, and PPDK complexes with nitazoxanide (NTZ) and the BCL compounds. The panels (**E**–**H**) illustrate the three-dimensional binding modes and ligand overlapped at catalytic sites, interacting with essential cofactors (represented as spheres), highlighting key hydrogen bonds, electrostatic contacts, and hydrophobic interactions.

To further evaluate the docking predictions, the highest-ranked ligand–enzyme complexes were subjected to 250 ns all-atom molecular dynamics (MD) simulations. Complex stability was assessed using the Cα root mean square deviation (RMSD) trajectories (Figure 9A–D) together with representative 3D interaction snapshots for NTZ and the selected CLB compounds. All simulated systems reached relatively RMSD plateaus during the trajectories. The G6PD::6PGL-CLB-3, PFOR-CLB-3, PK-CLB-5, and PPDK-CLB-5 complexes generally stabilized within the first 20–40 ns and exhibited lower global fluctuations than the corresponding NTZ complex. These results suggest that CLB-3 and CLB-5 remain more consistently accommodated within the modeled binding regions. In contrast, NTZ showed greater positional variability, particularly in the PK and PFOR systems, indicating less stable retention within the evaluated pockets. In the G6PD::6PGL complex, CLB-3 maintained persistent contacts with residues surrounding the NADP^+^-binding region, including interactions near the β-phosphate cleft and constrained local loop motions (Figure 9E). This orientation may interfere with cofactor accommodation or local conformational changes required for catalysis. CLB-3 also remained positioned between ATP and the CoA access channel, physically preventing catalytic of the phosphate groups and electron transfer (Figure 9G).

In the PK complex, CLB-5 formed persistent hydrogen bonds and electrostatic interactions near the magnesium ion (Mg^2+^) and adenosine triphosphate (ATP)-associated catalytic region (Figure 9F). The predicted pose placed the compound close to residues involved in the recognition of phosphorylated substrates and cofactors. This arrangement is compatible with possible interference in the catalytic environment required for the conversion of phosphoenolpyruvate (PEP) to pyruvate (Figure 9F). In contrast, within PPDK, CLB-5 showed exceptional stability inside the ATP-binding pocket, forming long-lasting polar interactions with amino acid residues responsible for transferring phosphate groups using ATP (Figure 9H). Finally, in simulations in which NTZ was bound, the compound remained less stably anchored in all four enzyme pockets. It frequently shifted toward the solvent interface or lost contact with essential catalytic residues, which aligned with its more peripheral binding positions and lower ability to inhibit the enzymes. Taken together, these molecular findings suggest that CLB-3 and CLB-5 could act as multi-target inhibitors. The prediction that they could simultaneously destabilize essential glycolytic enzymes (PK), alternative pathways (PPDK, PFOR), and the pentose phosphate pathway (G6PD::6PGL) explains the potent metabolic effect observed experimentally and justify their therapeutic efficacy even in NTZ-resistant *G. lamblia* strains.

**Figure 10:**
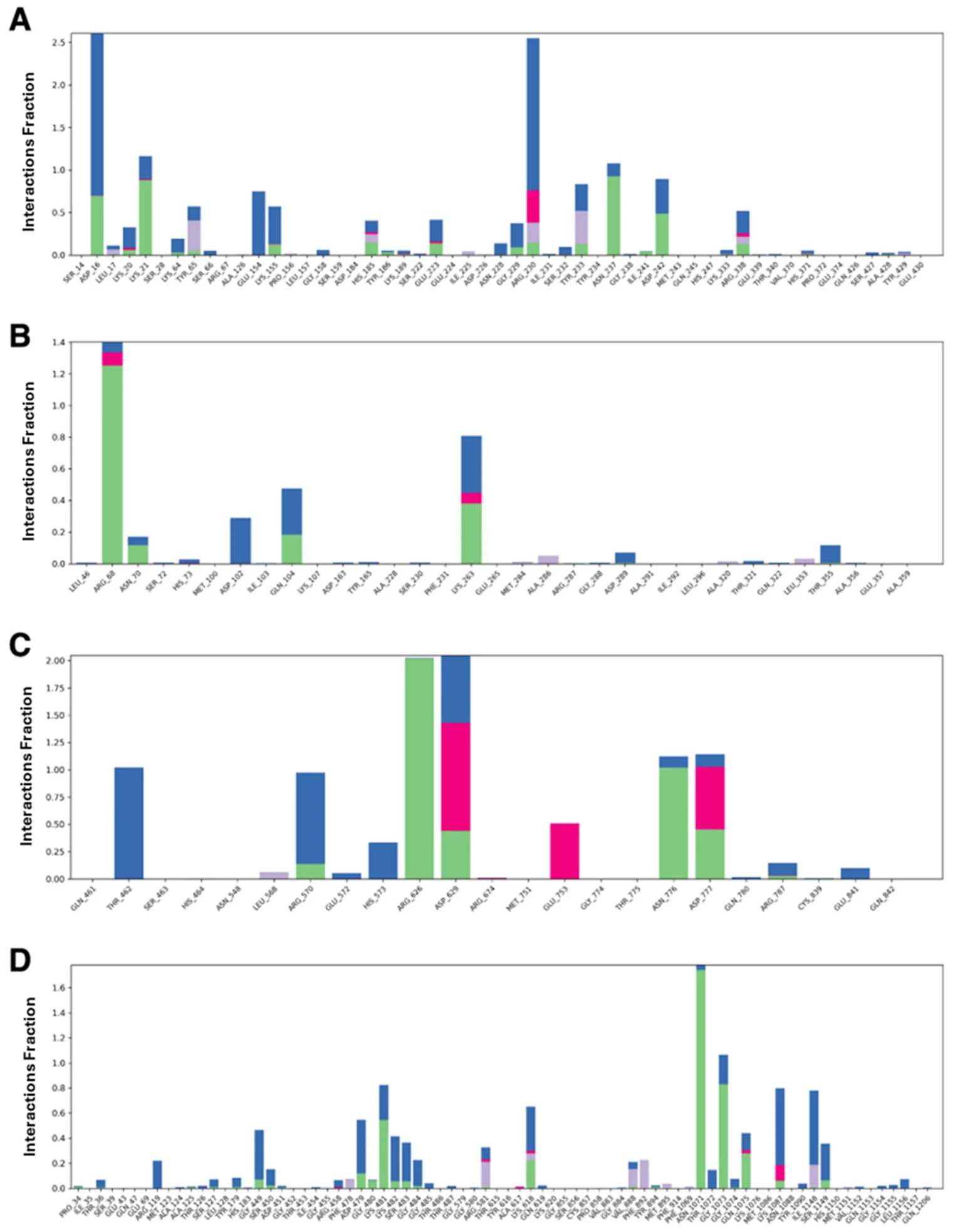
MD interaction frequencies reveal stronger and more persistent contacts for CLB derivatives of (**A**) G6PD:6PGL, (**B**) PK, (**C**) PFOR, and (**D**) PPDK. Interaction frequency maps display the persistence (%) of hydrogen bonds (green), hydrophobic contacts (purple), π–π interactions (blue), and charged contacts (pink) across the trajectories. Each panel details the *Giardia lamblia* enzyme residues that consistently interact with the evaluated compounds.

To determine which amino acids stabilize CLB-3 and CLB-5 within the binding sites of each protein target, ligand–protein interaction fractions were calculated during the 250 ns of molecular dynamics. Figure 10 shows the residue-level interaction profiles from the MD simulations for NTZ compared with CLB-3 (G6PD::6PGL, PFOR) and CLB-5 (PK, PPDK). CLB-3 consistently displayed higher contact frequency with catalytic residues. It also showed greater hydrogen-bond occupancy, broader interaction surfaces, and strong engagement of cofactor-binding motifs (NADP^+^ in G6PD::6PGL; TPP/CoA in PFOR). For the G6PD::6PGL enzyme, the contacts with Asp14 and Arg230 are noteworthy, as they exhibited very high interaction fraction values (greater than 2.5), (Figure 10A). The Asp14 residue is a conserved component in G6PD proteins and corresponds to the nucleotide-binding fingerprint (GxxGDLA). Its peptide has been associated with the binding of the coenzyme NADP^+^ located between amino acids 12 and 18 at the N-terminal end of the G6PD::6PGL protein [39]. The fact that CLB-3 maintains such strong interactions (mainly mediated by water and hydrogen bonds) with this residue suggests that the mechanism of action involves a direct steric block in the reduction of NADP^+^ to NADPH, which would generate a probable inactivation of the pentose phosphate pathway, leading to a decrease in the production of pentoses for nucleic acid synthesis and NADPH as the principal modulator of intracellular redox potential. On the other hand, the PFOR complex with CLB-3 (Figure 10C) shows interactions in the vicinity of the coenzyme A (CoA) recognition site. The terminal residues Asn1071, Gly1073, and Gly1074 form hydrogen bonds. In the PFOR complex, the CoA binding channel and the anchoring of its phosphate groups depend critically on glycine-rich peptide loops, which provide the necessary flexibility and space for the cofactor [50]. Blocking this C-terminal region with CLB-3 disrupts CoA’s access to the iron-sulfur cluster 4Fe-4S, preventing the oxidative decarboxylation of pyruvate.

The compound CLB-5 showed a similar trend for PPDK and PK; it interacted robustly with residues governing ATP-dependent phosphate transfer and PPi-mediated catalysis. For the PK complex with CLB-5 (Figure 10B), the interaction is concentrated at the Arg68 residue (fraction > 1.2), stabilized by direct hydrogen bonds and electrostatic contacts. In *G. lamblia* PK, Agr68 directly coordinates the negatively charged phosphate groups of the substrate [51]. In the PPDK complex with CLB-5 (Figure 10D), residues Arg626 and Asn776 maintained stable hydrogen bonds throughout the simulation, while amino acids Asp629 and Asp777 maintained strong ionic interactions with the compound. In the molecular architecture of PPDK, these charged polar residues are an essential part of the C-terminal region, as they bind and stabilize pyruvate and phosphoenolpyruvate [52]. Therefore, the persistent occupation of these residues by CLB-5 competitively inhibits PPi-dependent ATP resynthesis. NTZ exhibited fewer, less stable interactions, consistent with its higher IC_50_ values and weaker binding stability. Overall, CLB-3 and CLB-5 display an interaction fingerprint typical of multi-target, catalysis-disrupting inhibition. Molecular contact histograms suggest that CLB-3 and CLB-5 do not bind non-specifically to proteins but rather may act as targeted inhibitors by binding to catalytic residues and essential cofactor-binding sites such as Arg230 (G6PD::6PGL), Arg68 (PK), Arg626 (PPDK), and the C-terminal glycine loop (PFOR). This could also explain the coordinated collapse of the parasite’s energy metabolism observed experimentally.

The computational results provide a structural rationale for biological observations. CLB-5 is highly potent, matching its deep, stable binding to pyruvate phosphate dikinase (PPDK), a *G. lamblia*-specific enzyme essential for ATP generation. CLB-3 binds strongly to three major metabolic enzymes at once: glucose-6-phosphate dehydrogenase (G6PD), pyruvate kinase (PK), and pyruvate:ferredoxin oxidoreductase (PFOR). This multi-target action helps explain CLB-3’s strong IC_50_ values and broad transcriptional changes. Stronger catalytic-site engagement by CLB compounds correlates with the downregulation of glycolytic genes—phosphofructokinase (PFK), PK, and PPDK—and changes in redox-regulation pathways. Taken together, these findings support a model: CLB-3 and CLB-5 act as multi-target metabolic disruptors. They surpass NTZ in both binding affinity and catalytic interference. This explains their high activity against NTZ-susceptible and NTZ-resistant *G. lamblia* strains.

### 2.7. Study Limitations

Despite the promising antigiardial results obtained in this study, significant methodological and interpretative limitations remain that must be considered. First, biological evaluations were strictly limited to in vitro assays and in silico computational simulations; consequently, the therapeutic efficacy, pharmacokinetic behavior, and systemic toxicity of the derivatives in in vivo models have not yet been determined. Second, although molecular docking and dynamics models suggest selective affinity for key enzymes, direct physical binding and actual enzymatic inhibition have not been experimentally validated at the biochemical level. Finally, changes observed in the gene expression profile do not automatically translate into a functional metabolic blockade; therefore, direct measurements of enzyme activity and glucose consumption are required to conclusively confirm the proposed mechanism of action. Although molecular docking and dynamics models predict high affinity and stability with key enzymatic targets (such as G6PD::6PGL, PK, PPDK, and PFOR), direct physical binding and actual enzymatic inhibition have not been experimentally validated using purified proteins.

## 3. Materials and Methods

### 3.1. Strains and Cell Lines

This study used two *G. lamblia* strains: a nitazoxanide-susceptible strain from the American Type Culture Collection (ATCC 30957), provided in collaboration with Hospital Infantil de México Federico Gómez, and a nitazoxanide-resistant strain from PhD. Ignacio de la Mora-De la Mora at the Instituto Nacional de Pediatría. The Caco-2 cell line was obtained from Luz Maria Rocha Ramirez from Hospital Infantil de México Federico Gómez, Secretaría de Salud.

### 3.2. Determination of IC_50_ of CLB Compounds in Giardia lamblia Strains

The antigiardial activity of seven synthetic compounds belonging to the CLB series was evaluated. These compounds were selected from an in-house library of 120 organic molecules and were kindly provided by Dr. Gabriel Navarrete-Vázquez (Autonomous University of the State of Morelos). The CLB series comprises nitrothiazole-clofibric acid chimeras that share a common structural scaffold (Figure 1). Their synthesis and chemical characterization have been previously described [16,17,18,19,22]. A viability assay was carried out on *G. lamblia* strains ATCC 30957 (NTZ-sensitive) and an NTZ-resistant strain. Strains were cultured in TYI-S-33 medium with 10% fetal bovine serum and antibiotics in 8 mL screw-cap tubes, inoculated with 2 × 10^5^ trophozoites, and incubated at 37 °C for 48 h or until confluence. After incubation, trophozoites were collected and counted as described by Hernández-Ochoa et al. [53]. For treatment, 1.5 × 10^5^ trophozoites were placed in 1.5-mL microcentrifuge tubes with supplemented TYI-S-33 medium and exposed to various CLB concentrations (0–25 µM). A tube without compound served as a negative control, and nitazoxanide (NTZ) was used as a positive control. All samples were incubated at 37 °C for 48 h, then cell viability was assessed by trypan blue staining [20,53]. Viability for each compound was compared to the control, and IC_50_ values were calculated using the online IC_50_ Calculator (AAT Bioquest, https://www.aatbio.com/tools/ic50-calculator) (accessed on 20 April 2026). IC_50_ data are expressed as the mean ± standard deviation (SD) from three independent experiments, each conducted with three replicates.

### 3.3. Gene Expression Analysis by RT-qPCR of Giardia lamblia Trophozoites Exposed to the Compounds

To evaluate the effect of CLB compounds on gene expression in NTZs and NTZr *G. lamblia*, trophozoites were exposed to the IC_50_ for each compound, and the expression levels of 14 genes involved in parasite metabolism were measured by RT-qPCR. The oligonucleotides used in this study have been previously reported [20,54,55].

From an inoculum of 1.5 × 10^4^ of *G. lamblia* trophozoite NTZs and NTZr (exposed for 48 h at 37 °C to the IC_50_ of compounds CLB-3 and CLB-5 and their respective vehicle controls), samples were collected by centrifugation at 800× *g* for 5 min at 4 °C. Total RNA was isolated using TRIzol^®^ reagent (Thermo Fisher Scientific, Waltham, MA, USA) according to the method reported by Gutiérrez-Cardona et al. [56]. To remove any residual genomic DNA, the samples were treated with DNase I (Thermo Fisher Scientific, Waltham, MA, USA). Then, RNA concentration and purity were determined spectrophotometrically by measuring the absorbance ratio at 260/280 nm using a NanoPhotometer^®^ spectrophotometer (IMPLEN, Westlake Village, CA, USA). Only samples with a ratio between 1.8 and 2.0 were used for subsequent steps. RNA integrity was visually verified by electrophoresis on a 2% agarose gel stained with GelRed. The synthesis of the first complementary DNA (cDNA) strand was performed using the Oligo dT18 technique and reverse transcriptase (Thermo Fisher Scientific, Waltham, MA, USA), following the protocol reported by Hernández-Ochoa et al. [57].

Real-time PCR reactions were performed using SYBR™ Green Master Mix (Thermo Fisher Scientific) on an Applied Biosystems StepOne™ real-time PCR system. Reactions were prepared in a final volume of 20 µL, containing the Master Mix, forward and reverse primers (200 nM), and 100 ng of cDNA. The amplification program consisted of an initial activation at 95 °C for 30 s, followed by 40 cycles of denaturation at 95 °C for 30 s, annealing at 60 °C for 30 s, and extension at 72 °C for 30 s. For normalization of the expression data, the fructose-1,6-bisphosphate aldolase gene was selected as an endogenous reference control, according to the transcriptional stability criteria previously described by Macial-Quino et al. [54]. The relative change in mRNA levels (fold-change) was calculated using the 2^−∆∆CT^ method.

### 3.4. Cytotoxicity Assessment of Chemical Compounds in Caco-2 Cell Cultures

To assess the cytotoxicity of CLB compounds, human Caco-2 cells (ATCC HTB-37) were exposed to them. The Caco-2 cell line was obtained from Luz Maria Rocha Ramirez from Hospital Infantil de México Federico Gómez, Secretaría de Salud. The assay was performed in 96-well plates, with 5 × 10^3^ cells inoculated into 100 mL of DMEM supplemented with 10% FBS and antibiotics in each well. The cells were exposed to different concentrations of the compounds for 48 h. Cell viability was then measured using the Cell Proliferation Kit II (XTT; St. Louis, MO, USA) according to established methods [20,52]. Finally, CC_50_ values were calculated using nonlinear regression in GraphPad Prism version 8 for Windows. CC_50_ data are expressed as the mean ± standard deviation (SD) from three independent experiments, each conducted with three replicates.

### 3.5. Quantification of ATP, ADP, and the ADP/ATP Ratio

To assess the cellular bioenergetic status of trophozoites treated with BCL-3 and BCL-5, a cell suspension containing 1.5 × 10^5^ trophozoites/1.5 mL of culture medium was prepared, and 100 µL aliquots were seeded into the wells of 96-well microplates (with opaque white bottoms). The parasites were exposed to the previously determined IC_50_ of the compounds. Negative control wells (trophozoites exposed only to DMSO) and background control wells (culture medium without cells) were included simultaneously to correct for interference. The plates were incubated at 37 °C for 48 h. Intracellular ATP and ADP levels were quantified using the commercial ADP/ATP Ratio Assay Kit (Cat. No. MAK135, Sigma-Aldrich, Darmstadt, Germany), strictly following the manufacturer’s protocol. Briefly, after 48 h of incubation, ATP Reagent was added to each well. Following a 10-min incubation at room temperature, initial luminescence was measured using a microplate reader. This signal is directly proportional to the basal ATP concentration. Immediately thereafter, the ADP reagent was added. After an additional 10-min incubation at room temperature, a second luminescence measurement was performed.

### 3.6. Statistical Analysis

All experiments were carried out in three independent experiments performed with three replicates each. The data are presented as the mean ± standard deviation (SD). For the antigiardiasis activity and cytotoxicity assays, the absorbance readings obtained with the XTT reagent were converted to percentages of inhibition of parasite growth and cell viability in Caco-2 cells, respectively. The inhibitory media concentrations (IC_50_) and the cytotoxic media concentrations (CC_50_) were calculated by non-linear regression analysis. The selectivity index (IS) was calculated independently for each compound using the equation: IS = CC_50_ in Caco-2 cells/IC_50_ in *G. lamblia* trophozoites. Gene expression data and relative change values obtained by 2^−∆∆CT^ were analyzed by one-way analysis of variance (ANOVA), followed by the Mann–Whitney test, to determine significant differences between the treated and untreated trophozoites. All statistical analyses and graphical representations were performed using GraphPad Prism version 8.0 (GraphPad Software Inc., La Jolla, CA, USA), considering a *p*-value < 0.05 as statistically significant.

### 3.7. Molecular Simulations Methodology

#### 3.7.1. Protein Structure Preparation

Five *G. lamblia* metabolic enzymes were selected as potential candidates for antigiardiasic activity. The enzymes were chosen as molecular targets due to their essential roles in glycolysis and redox homeostasis. The selected molecular targets included glucose-6-phosphate::6-phosphogluconate dehydrogenase fused enzyme (G6PD::6PGL), pyruvate kinase (PK), pyruvate phosphate dikinase (PPDK), and pyruvate:ferredoxin oxidoreductase (PFOR). Because all targets lack experimentally resolved structures, we predicted their three-dimensional (3D) structures with AlphaFold3 *via* the publicly available *AlphaFold server* (https://alphafoldserver.com, accessed 5 November 2025). AlphaFold3 is optimized for speed and accessibility [58]. Moreover, relaxation cycles were applied to improve sidechain packing and reduce steric clashes in the final models. The model with the highest *pLDDT* (predicted local distance difference test) score was selected for further analyses. To assess model quality, we used *per*-residue *pLDDT* scores and predicted aligned error (PAE) plots. Visual inspection and structure rendering were performed with Maestro v. 2024-4 (Schrödinger, LCC. New York, NY, USA) to examine global folds and secondary structure elements. Multiple cofactors—thiamine pyrophosphate, [4Fe-4S] clusters, and coenzyme A (PFOR); ATP, ADP, Mg^2+^, Mn^2+^ (PK and PPDK); and NADH^+^ (G6PD::6PGL)—were added to the models using Alphafill [58]. The fit of the inserted groups was optimized using the Alphafill web server to achieve transplant clash score (TCS) values below 0.29, which is considered a high-confidence fit.

#### 3.7.2. Ensemble Docking Calculations

The molecular dockings of the CLB library (ligands) to the putative active site of protein targets (receptors) were performed using the GNINA suite (v. 1.3.2) for CUDA 12.8 [59]. This molecular docking tool integrates both traditional scoring functions and deep learning-based scoring models to enhance pose prediction and binding affinity assessment [59,60]. Representative enzyme conformations (ten conformations) were selected from the initial 100 ns molecular dynamics trajectories using hierarchical alpha carbon (C⍺) RMSD-based clustering implemented in Desmond (~3 Å of the centroid). The centroids of the most populated clusters were used as the representative receptor conformation because they correspond to the dominant structural state sampled during the simulation. This procedure reduces bias introduced by a single static model structure while preserving the most probable active-site geometries. The selected conformations were prepared for docking using the Protein Preparation Workflow routine in Maestro v. 2024-4. Protonation states of titratable residues were assigned at pH 7.4 using PROPKA3 [61], and hydrogen-bond networks were optimized. All CLB ligands (CLB-1 to CLB-9) and nitazoxanide (NTZ) were sketched, and each structure was energy-minimized with OPLS-AA [62,63]. The lowest-energy conformer of each ligand was used for ensemble docking.

GNINA was executed with Monte Carlo exhaustivity set to 128, after which CNN scoring was applied to refine pose ranking using deep convolutional neural network predictions. The receptor chemical search space was defined by the known or predicted binding pocket. Subsequently, a 20 × 20 × 20 Å box was centered on each binding site to allow ligand conformational flexibility.

For each molecular docking run, GNINA generated 10 binding poses per ligand-receptor complex, from which the top poses were selected based on CNN affinity scores. Subsequently, graphs were generated using GraphPad Prism 10 (GraphPad Software, Boston, MA, USA).

#### 3.7.3. Molecular Dynamics Simulations

The most favorable CNN-docking score complexes were selected as initial coordinates for molecular dynamics simulations to identify stable CLB binding modes. All ligand-enzyme simulations were conducted with Maestro v. 2024-4 version 13.8.135, MMshare Version 6.4.135 Release 2023-4, and the Desmond Multisim v4.0.0 interoperability tools package, using the OPLS3 force field [64]. The system complexes were solvated in octahedral boxes of explicit TIP3P water molecules. These boxes extended 15 Å from the solute and were neutralized with 0.15 M Na^+^ or Cl^−^ ions. All MD simulations were carried out under periodic boundary conditions. Initial energy minimizations were performed using a two-approximation scheme: 10,000 steps of steepest descent followed by 10,000 steps of conjugate gradient. Positional restraints of 10 kcal/mol·Å^2^ were applied to the solute heavy atoms. The system was gradually heated from 0 to 310 K over 1000 ps under constant-volume (NVT) conditions. This was followed by 500 ps of equilibration at 310 K under constant-pressure (NPT) conditions with a relaxation time of 1 ps, using the Langevin thermostat. The Berendsen barostat maintained a pressure of 1 atm, with a relaxation time of 2 ps. Positional restraints were gradually reduced during equilibration. Conventional MD simulations (three replicas) were performed for 250 ns at 1 atm. These were performed using the Tobias Barostat at 310 K, with the Noose–Hover chain thermostat, Maestro v. 2024-4 version 13.8.135, MMshare Version 6.4.135 Release 2023-4, and Desmond Multisim v4.0.0 interoperability tools package (New York, NY, USA). Weak coupling adjusted the temperature to an external bath at 303 K. The Particle Mesh Ewald (PME) method computed long-range electrostatic interactions (tolerance of 1 × 10^−12^) within the periodic system. Integration times were set to 2 fs.

Trajectory analysis was conducted using the Simulation Event Module of the Desmond-Maestro interoperability tools. Alpha carbon root mean square deviation (RMSD, a measure of protein backbone movement over time) was calculated along complete trajectories to assess structural flexibility, and fractions of intermolecular interactions were calculated for each CLB compound.

## 4. Conclusions

This study demonstrates the significant potential of new organic derivatives (particularly CLB-3 and CLB-5) as therapeutic candidates against *Giardia lamblia*, as they exhibit high in vitro antigiardial potency and remain effective against both susceptible strains and the nitazoxanide-resistant (NTZr) strain. Furthermore, the compounds showed low cytotoxicity in Caco-2 cells, yielding promising selectivity indices that support their preliminary biological safety. The key identity lies is the identification—through transcriptomic analysis and in silico simulations—of a multifactorial mechanism of action linked to the disruption of redox homeostasis and the blockade of the glycolytic pathway via key enzymatic targets (such as G6PD::6PGL, PK, PPDK, and PFOR). Thus, this research not only provides robust scientific alternatives to address the growing therapeutic failure of conventional nitroimidazoles, but also establishes the molecular foundation necessary for the future development of safer and more effective antiparasitic therapies.

## Figures and Tables

**Table 1:** IC_50_ values of synthetic CLB compounds against trophozoites of the nitazoxanide-susceptible (NTZ-s) *G. lamblia* ATCC 30957 strain and an NTZ-resistant *G. lamblia* strain.

	IC_50_ (µM)		Resistance Index	Caco-2	Selectivity Index	
Compound	*G. lamblia* NTZs (Relative Potency to NTZ)	*G. lamblia* NTZr (Relative Potency to NTZ)		CC_50_ (µM)	Relative NTZs	Relative NTZr
CLB-1	0.40 ± 0.02 (10-fold)	0.36 ± 0.02 (45-fold)	0.9	ND	ND	ND
CLB-3	0.15 ± 0.01 (27-fold)	0.17 ± 0.01 (42-fold)	1.13	>500	>3333	>2940
CLB-4	0.28 ± 0.02 (15-fold)	0.13 ± 0.007 (56-fold)	0.46	ND	ND	ND
CLB-5	0.07 ± 0.004 (60-fold)	0.12 ± 0.01 (60-fold)	1.71	310	4428	2583
CLB-6	0.21 ± 0.01 (19-fold)	0.56 ± 0.03 (13-fold)	2.6	>500	>2380	>892
CLB-7	0.28 ± 0.02 (15-fold)	0.38 ± 0.02 (19-fold)	1.35	>500	>1785	>1315
CLB-9	1.7 ± 0.10 (2.5-fold)	1.07 ± 0.05 (6.7-fold)	0.62	ND	ND	ND
NTZ	4.22 ± 0.21	7.24 ± 0.40	1.71	580 [20]	137	80

IC_50_ data are expressed as the mean ± standard deviation (SD) from three independent experiments, each conducted with three replicates. Values in parentheses indicate the fold increase in antigiardial activity relative to NTZ. Values in square brackets indicate the selectivity index (SI), calculated as the ratio of the CC_50_ in mammalian cells to the IC_50_ against trophozoites of the *G. lamblia* ATCC strain 30957 (SI = CC_50_/IC_50_). ND = not determined.

## Data Availability

The original contributions presented in this study are included in the article/Supplementary Material. Further inquiries can be directed to the corresponding authors.

## Supplementary Materials

The following supporting information can be downloaded at: https://www.mdpi.com/article/10.3390/molecules31172980/s1, Table S1. CNN-docking scores (kcal·mol^−1^) of nitazoxanide (NTZ) and CLB derivatives against four *Giardia lamblia* metabolic enzymes obtained by ensemble docking.

## Abbreviations

The following abbreviations are used in this manuscript:

*ACT*	Actin
*ALDO*	Aldolase
IC_50_	Inhibition concentration 50
*GIA*	Giardin
*GAPDH*	Glyceraldehyde-3-phosphate dehydrogenase
*G6PD*::*6PGL*	Glucose-6-phosphate dehydrogenase::6-phosphogluconolactone
*6PGDH*	6-Phosphogluconate dehydrogenase
LTS	Low-throughput screening
MD	Molecular dynamics
MTZ	Metronidazole
*NADH*ox	NADH-oxidase
NTZ	Nitazoxanide
NTZr	Nitazoxanide-resistant
NTZs	Nitazoxanide-susceptible
PEP	Phosphoenolpyruvate
*PK*	Pyruvate kinase
*PFK*	Phosphofructokinase
*PFOR*	Pyruvate-ferredoxin oxidoreductase
*PPDK*	Pyrophosphate-dependent pyruvate phosphate dikinase
PPP	Pentose phosphate pathway
RI	Resistance index
RMSD	Root mean square deviation
SD	Standard deviation
SI	Selectivity index
*TKT*	Transketolase
*TPI*	Triosephosphate isomerase
*VSP*	Variant-specific surface proteins
