# Supplementary material for: 5-Nitrothiazol-Clofibric Acid Analogs as Potent Agents Against Nitazoxanide-Resistant Giardia lamblia: Modulation of Glycolytic Genes and Molecular Dynamics Studies
Source: Molecules. 2026 Aug 26;31(17):2980. doi: 10.3390/molecules31172980 (PMC13567709; doi:10.3390/molecules31172980)
Supplement: Supplementary file 1 [file molecules-31-02980-s001.zip › molecules-4471758-supplementary.pdf]

5-Nitrothiazol-Clofibric Acid Analogs as Potent Agents Against Nitazoxanide-Resistant Giardia lamblia: Modulation of Glycolytic Genes and Molecular Dynamics Studies

Beatriz Hernández-Ochoa [1], Blanca Colin-Lozano [2], Gabriel Navarrete-Vázquez [3], Sergio Enríquez-Flores [4], Ignacio De la Mora-De la Mora [4], Verónica Pérez de la Cruz [5], Roberto Arreguin-Espinosa [6], Rosa Angélica Castillo-Rodríguez [7], José Antonio Velázquez-Aragón [8], Julieta Griselda Mendoza-Torreblanca [9], Noemí Cárdenas-Rodríguez [9], Daniel Ortega-Cuellar [10], Cindy Bandala [11], Abraham Vidal-Limon [12,*], and Saúl Gómez-Manzo [13,*]

Table S1. CNN-docking scores (kcal·mol^{-1}) of nitazoxanide (NTZ) and CLB derivatives against four *Giardia lamblia* metabolic enzymes obtained by ensemble docking.

**Compound**	**G6PD::6PGL (kcal·mol^{-1})**	**PK (kcal·mol^{-1})**	**PPDK (kcal·mol^{-1})**	**PFOR (kcal·mol^{-1})**
**NTZ**	−4.03 ± 1.63	−5.50 ± 0.59	−6.51 ± 0.52	−5.38 ± 0.51
**CLB-1**	−0.84 ± 1.26	−5.64 ± 0.58	−6.65 ± 0.63	−6.07 ± 0.77
**CLB-3**	**−3.96 ± 1.19**	**−5.95 ± 0.56**	**−7.64 ± 0.84**	**−6.45 ± 0.53**
**CLB-4**	−3.68 ± 1.81	−5.45 ± 0.46	−6.58 ± 0.63	−5.65 ± 0.44
**CLB-5**	**−3.84 ± 1.08**	**−5.88 ± 0.73**	**−7.06 ± 0.79**	**−6.42 ± 0.53**
**CLB-6**	−3.86 ± 1.76	−5.63 ± 0.48	−6.83 ± 0.76	−6.03 ± 0.50
**CLB-7**	−1.38 ± 2.13	−5.77 ± 0.68	−6.96 ± 0.68	−6.08 ± 0.65
**CLB-9**	−2.81 ± 2.77	−5.63 ± 0.72	−7.20 ± 0.99	−6.21 ± 0.66

* Values are expressed as the mean ± standard deviation from docking calculations performed using ten representative receptor conformations extracted from 100 ns molecular dynamics simulations.
